# The survey of soil-transmitted helminth species abundance in Slovakia with an emphasis on parameters important for their distribution

**DOI:** 10.3389/fmed.2022.1043313

**Published:** 2022-11-17

**Authors:** Lukáš Ihnacik, Júlia Šmigová, Jindřich Šoltys, Diana Bobíková, Žofia Kuzevičová, Štefan Kuzevič, Ingrid Schusterová, Ingrid Papajová

**Affiliations:** ^1^Institute of Parasitology, Slovak Academy of Sciences, Košice, Slovakia; ^2^Department of Public Veterinary Medicine and Animal Welfare, University of Veterinary Medicine and Pharmacy in Košice, Košice, Slovakia; ^3^Faculty of Mining, Ecology, Process Control, and Geotechnologies, Technical University of Košice, Košice, Slovakia; ^4^Faculty of Medicine, East Slovak Institute of Cardiovascular Diseases, Košice, Slovakia

**Keywords:** soil-transmitted helminths, people, dogs, soil, epidemiology, environmental factors

## Abstract

Soil-transmitted helminths (STH) can be easily dispensable in socially disadvantaged groups. The Roma people represent the group most at risk in Slovakia. This study aimed to investigate the occurrence of STH infections in minorities living with animals under low hygienic conditions and on contaminated soil. Subsequently, we identified the risk assessment factors of the STH transmissions based on parasitological results. In our study, STHs were predominantly found among the Roma communities living in unsanitary conditions. The prevalence of *Ascaris lumbricoides* among the majority was 0.79%, and *Trichuris trichiura was* 0.05 %. On the contrary, a community-based cross-sectional survey across eastern Slovakia also found a prevalence of 22.28 and 3.47% for *A. lumbricoides* and *T. trichiura* among the Roma population. Inhabitants that belong to the Roma minority had a 37.12 infection OR times higher than non-Roma inhabitants. The Roma people living in the countryside have a 2.23-fold higher chance of getting infected with STH than Roma living in the city. Therefore, soil and domestic animals were also examined for the presence of the STH eggs to show the environmental burden. In general, the presence of STH eggs was confirmed in 26.26% of the soil examined samples. The detailed description is as follows: eggs of *Toxocara* spp., *Trichuris* spp., eggs from the family Ancylostomatidae, and *Toxascaris leonina*. *Ascari*s eggs were detected only in the soil from localities with low hygienic standards. The probability of contracting the STH eggs in segregated settlements was 15.94 times higher compared to urban or rural areas. In addition to humans, dogs can also be a source of STH eggs in the soil. The STH eggs were confirmed in 43.55% of dog droppings. The most interesting finding was that the eggs of the genus *Ascaris* were up to 7.93% of dog droppings from localities with a low hygienic standard were positive. This study revealed that climatic factors and the WASH conditions influenced the distribution of STHs to variable degrees. In addition, ethnicity and sanitation were crucial factors in the distribution of STH infection in eastern Slovakia.

## Introduction

Soil-transmitted helminths (STH) represent the most prevalent parasitic infections worldwide and belong to the most spread neglected diseases. However, STH can be easily dispensable in socially disadvantaged groups. So primarily, these diseases occur in the population of marginalized communities, which are distinct by complete social exclusion from the major population, where consequences of various epidemiological and environmental factors take place. The STH is inextricably linked to poverty, inadequate sanitation, poor hygiene conditions, and social behavior. In Slovakia, the group most at risk is represented by the Roma people living in Eastern Slovakia. The Roma population has a progressive age structure, i.e., a high proportion of the younger population and a low proportion of the population over the age of 60. Life expectancy, considered a fundamental indicator of the population’s health status, is in Roma men and women only 55.3 or 59.5 years, respectively. WHO specifies the same numbers for life expectancies in developing countries (1–5). Roma predominantly lives in segregated settlements and communities isolated from places where the majority population lives. Segregated settlements present the worst living scenario regarding the existence of low personal and municipal hygiene. Low living standards are linked with poor infrastructure, where just 10.0% of settlements have limited access to the main paved roads. Regarding the standard utility infrastructure in the Roma settlements, electricity is most available (98.0% of settlements). The least available is public waste management, as only 40% of these settlements have a public sewer system. Gas is unavailable in 55% and water pipes in 21% of the settlements. Public water supply is used only in 64% of segregated communities. Disturbing is the reality that up to 8% of dwellings do not have direct access to drinking water (6). Drinking water is most often taken from natural sources such as streams or creeks and consumed regularly without proper sanitary treatment. The Roma people live in shelters, on the outskirts of towns and villages, or in the forest’s vicinity. They are exposed much more to wildlife (rodents, birds, foxes, stray cats, and dogs) and other potential biological agents allowing the spread of infectious diseases (mosquitoes, ticks, flies, etc.). At the same time, in a small area, people live together with domestic animals, often without proper veterinary management and supervision. Due to this lack of veterinary care, infrastructure, and less frequent waste removal, feces from stray dogs accumulate in settlements and human feces, as it is a common phenomenon to defecate near houses and streets. Thus, representing a significant health risk for the circulation of soil-transmitted infections between animals and man. The non-Roma population, on the other hand, lives in entirely different conditions and has a higher quality of hygiene with proper veterinary control of domestic animals. The lower concentration of inhabitants in individual dwellings, integration closer to the center of villages, and improved utility infrastructure (more than 84% of non-Roma inhabitants discharge wastewater into sewers or septic tanks, and more than 93% use public water supply) contribute to better sanitary conditions (6). But urban agglomerations are also distinct by the accumulation of public waste. Containers with collected garbage become a food source for rodents, birds, stray animals, and the socially underprivileged population. The studied regions are geographically and demographically comparable. The Košice and Prešov regions represent 32.1% of the state territory, where 1 591 147 inhabitants represent 29.2% of the Slovak citizens. The population density in the Košice region is 115.82 people/km^2^, and in the Prešov region, 91.96 people/km^2^. The size of the Roma community living in this territory is 240,882, which is one of the highest in Slovakia (6, 7). Even though the non-Roma population is frequently divided and secluded from the Roma community, these individuals share shared spaces with the majority, such as public transportation, retail centers, and health facilities. This allows for STH to be transmitted to the majority of the population as they come into contact with infectious stages present in soil contaminated with human feces from infected individuals. In contrast, human-to-human transmission is not so common in Slovakia. Therefore, environmental factors such as soil type, land surface temperature, and WASH (water, sanitation, and hygiene) influence STH transmission potential.

Papajová et al. (8) state that STH is primarily found in the eastern Slovakia children’s population. In children aged 7 months to 18 years without clinical symptoms, the prevalence of *A. lumbricoides* was 27.4% and *T. trichiura* 2.3%. The occurrence of these helminths in children younger than 2 years indicates that STH transmission in the community is a prerequisite. This observation pointed out the occurrence of intestinal STH in humans, dogs, and soil from the given ethnic group. However, the previous work did not evaluate the risk areas based on various environmental factors influencing the spread of STH. For this reason, this study aimed to investigate the occurrence of STH infections in major (non-Roma) and minor (Roma) populations living with animals under low-hygienic conditions and on contaminated soil. Subsequently, we assessed and identified the risk factors for STH transmissions based on parasitological results.

## Materials and methods

### Study design

**FIGURE 1:**
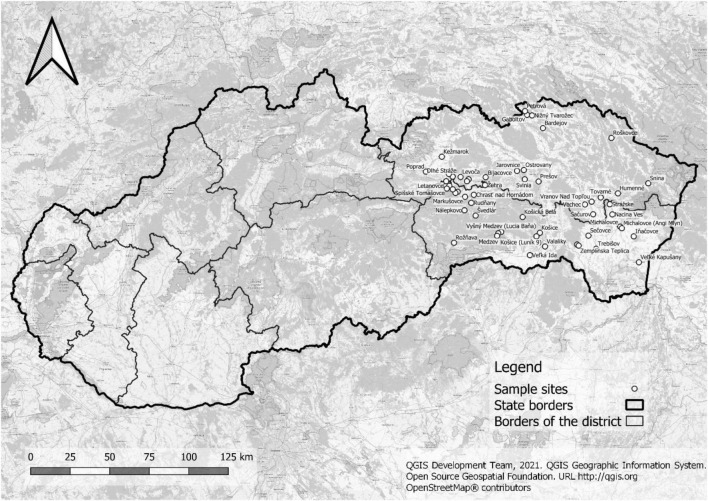
Studied localities.

The cross-sectional study was conducted in 17 towns, 29 villages, and 17 segregated settlements in eastern Slovakia (Figure 1). Stool, fecal, and soil samples were collected within the period from February 2018 up to May 2022. All samples were stored without any preservation at 4°C and immediately transported for further parasitological examination to the laboratory at the Institute of Parasitology of the Slovak Academy of Sciences in Košice. All analyses were performed within 24–48 h.

### Methods

#### Examination of stool samples

Overall, 4,841 stool samples were collected, and only one stool sample was obtained from each participant. Each sample specimen had to be supplemented with information about the participant’s age, gender, habitation, and minority or majority residency status. Samples that missed more than one of the above-collected criteria were excluded from the study. Approximately 20% of the samples (968/4841) had to be excluded from the total number and further analysis because of the small sample size or the lack of complete data. In the end, three thousand eight hundred seventy-three stool samples from eastern Slovakia inhabitants aged 0–80 years were examined. Participation was voluntary. Volunteers were divided into groups according to ethnicity (1,885 samples from non-Roma and 1,988 samples from Roma people). The epidemiological surveys were conducted with individuals, parents, guardians, and social workers working in segregated settlements. The participants in this study were clinically healthy, without any clinical signs of parasitic infection. After participants’ informed consent, including potential risks and benefits, was approved, stool samples were collected in plastic containers and marked with identification numbers along with the relevant data (residence, age, gender, and ethnicity). The stool sample volume was about 10–15 g for each individual. Samples for the presence of proglottids or possibly adult worms were at first examined macroscopically and subsequently assayed either by a commercially available kit (Paraprep L; Mondial, France) or with the SAF concentration method (sodium acetate, acetic acid, and formalin) (8). Paraprep L is a single-use, disposable device offering prevention against cross-contamination. The tangential hexagonal filter provides a reliable filtration of the sample. The preparation was carried out according to the manufacturer’s instructions. Briefly, 0.5 g of stool samples was placed on the filter and mixed in a tube with 2 ml of ethyl acetate and 6 ml of 10% formalin. Next, the tube was connected to a conical collection tube with a filter in the middle. After 24 h of incubation at room temperature, the tube is centrifuged at 500 x *g* for 1 min, the supernatant discarded, and the sediment examined microscopically with a Leica DM 5000B light microscope (Leica Microsystems; Wetzlar, Germany) at 100x and 400x magnification. The SAF concentration technique was also used for the detection of helminth eggs. In brief, ∼1 g of feces was used for examination. The SAF-fixed stool samples were re-suspended and strained through a medical gauze into a centrifuge tube. After centrifugation at 500 x *g* for 1 min, the supernatant was decanted, and the sediment was re-suspended in 7 ml of 0.85% NaCl. After adding 2–3 ml diethyl ether, the tube was closed with a rubber stopper, shaken vigorously for ∼30 s, and then centrifuged at 500 x *g* for 5 min. The three top layers were discarded, and the resulting sediment was examined microscopically with a Leica DM 5000B light microscope (Leica Microsystems; Wetzlar, Germany) at 100x and 400x magnification for the presence of helminths (8). The ethical commission reviewed and approved the study proposal (Document no. 5436/2019/ODDZ-25820).

#### Examination of dog’s excrements

2,836 dog fecal samples were collected and examined for the presence of STH eggs. Since the samples were collected randomly from public spaces near human dwellings, obtaining data on dogs’ age, sex, breed, eating habits, or health status was impossible. Although the sampling was carried out after each one at different locations, it was almost impossible to avoid duplicates due to the randomness of the collection. The research related to animals complied with all the relevant national regulations and institutional policies for the care and use of animals. Therefore, no additional authorization was required. A flotation method with Shaeter’s flotation solution of sucrose (specific gravity 1.27) was used for coprological examination of the excrements where 3 g of fecal sample was mixed with water and centrifuged for 5 min at 1,200 rpm (Eppendorf 5804, Hamburg, Germany). After pouring off the supernatant, Shaeter’s flotation solution was added to 2/3 of the tube. The mixture was mixed with the sediment and then centrifuged one more time. After 5 min, the test tube was refilled with flotation solution (until a meniscus formed) and covered with cover glass. After 1 h of egg flotation, the coverslip was removed and placed on the glass slide. All samples were examined under the light microscope at 200x and 400x magnification (Leica Microsystems, DM 5000B light microscope, Wetzlar, Germany).

#### Examination of soil samples

One thousand two hundred forty-nine soil samples were collected near human settlements and around the kennels and dog pens to identify the presence of parasites in the environment. However, it was often impossible to collect soil samples from the countryside and segregated settlements due to the owner’s reluctance and the compaction of the soil. The samples were surveyed according to Kazacos (9). Briefly, 100 g of the pooled sand sample, 100 ml of water, and 0.5 ml of Tween 40 were mixed and decanted for 10 min. Subsequently, the samples were sieved and replenished with 1,000 ml of water. After 1 h of sedimentation, the soil samples were centrifuged (Eppendorf 5804, Hamburg, Germany) and floated afterward with a sucrose flotation solution (specific density of 1.3). Samples were examined under the light microscope at 200× and 400× magnification (Leica Microsystems, DM 5000B light microscope, Wetzlar, Germany).

### Environmental, climatic, and demographic data

Environmental, climatic, and demographic data were collected within the period from February 2018 up to May 2022. The investigated localities are located in a temperate climate, where the basin and lowland cold/warm weather prevail. The average annual temperature ranges from 17 to 23°C. All studied areas’ average yearly rainfall precipitation ranged between 600 and 950 mm. Cambyses and fluvises soil types were the most abundant in the sites surveyed, although chernozems also occurred. The substrate consisted mainly of fluvial humic clays, clay loams, sandstones, sandy loams, sphagnum clays, and sphagnum loams. In all 57 selected locations in Košice and Prešov regions, the available demographic data analysis revealed that sewerage and drinking water were available for more than 80% of the inhabitants in 29 and 27 locations, respectively. On the other hand, only 11 sites used septic tanks for sewage disposal. The situation was different for the minority population, where 15 localities did not have access to clean drinking water, 26 did not have access to sewage, and 44 localities did not use septic tanks for waste disposal. The average rainfall data were obtained from the Eastern Slovak water company in Košice [Východoslovenská vodárenská spoločnost’, a.s., Košice, (10)]. Information about climate, average temperature, and soil type was acquired from the State geological Institute of Dionýz Štúr in Košice (11). Reports about drinking water, sewerages, and sumps in all studied localities were downloaded from Slovakia’s 2021 Population and Housing Census (7). Data covering marginalized communities (number of Roma, settlements, and presence of drinking water, sewerages, and sumps) were collected from the Atlas of Roma communities published in 2019 (6).

### Statistical analysis

Statistical analysis of the parasitological results was performed with STATISTICA (version 8.0; StatSoft, TIBCO Software; Palo Alto, CA, USA) software for data analysis and MS Excel (Office 365; Microsoft; Redmond, WA, USA). The odds ratio (OR) was calculated as the odds for the presence of STH eggs in samples from one group, which was calculated as the ratio between samples that contained STH eggs and samples without any STH eggs and subsequently divided by the odds of STH eggs presented in another group, in short, according to this equation (12):


$$O\,R=\frac{\begin{array}{c} oddsinonegroup\left(e.g.minority\right)\\ \left(\frac{n\,u\,m\,b\,e\,r\,o\,f\,p\,o\,s\,i\,t\,i\,v\,e\,s\,a\,m\,p\,l\,e\,s}{n\,u\,m\,b\,e\,r\,o\,f\,n\,e\,g\,a\,t\,i\,v\,e\,s\,a\,m\,p\,l\,e\,s}\right) \end{array}}{\begin{array}{c} oddsindifferentgroup\left(e.g.majority\right)\\ \left(\frac{n\,u\,m\,b\,e\,r\,o\,f\,p\,o\,s\,i\,t\,i\,v\,e\,s\,a\,m\,p\,l\,e\,s}{n\,u\,m\,b\,e\,r\,o\,f\,n\,e\,g\,a\,t\,i\,v\,e\,s\,a\,m\,p\,l\,e\,s}\right) \end{array}}$$

With OR calculated, we get an idea of which group or environment has the greater odds of contracting the diseases and by which parasite. After performing basic descriptive statistics (mean and standard deviation), the correlation coefficient was calculated according to Markechová et al. (12). The correlation coefficient was define


$$r=\frac{\sum_{i=1}^{n}\left(x_{i}-\bar{x}\right)\,\left(y_{i}-\bar{y}\right)}{\sqrt{\sum_{i=1}^{n}{\left(x_{i}-\bar{x}\right)}^{2}}\,\sqrt{\sum_{i=1}^{n}{\left(y_{i}-\bar{y}\right)}^{2}}}$$

In which:

*n:* is the size of the statistics file;

*x*: is the value of one character; $\bar{x}$*:* is the average of values of the x characters;

*y:* is the value of the second character; $\bar{y}$: average of the values of the y characters;

$\sqrt{\frac{1}{n}\,\sum_{i=1}^{n}{\left(x_{i}-\bar{x}\right)}^{2}}$: is the standard deviation of the characters *x* (σ_*x*_);

$\sqrt{\frac{1}{n}\,\sum_{i=1}^{n}{\left(y_{i}-\bar{y}\right)}^{2}}$: is the standard deviation of the characters *y* (σ_*y*_).

After calculating the correlation coefficient, we get values ranging from −1 to 1. Values approaching zero indicated little or no interdependence between the given factor and sample positivity. On the contrary, values approaching −1 or +1 revealed that there is an increasing dependence between the factor and sample positivity (12).

The correlation coefficient calculation converted this coefficient into a percentage value. This, which we used as an indicator to determine the weights for each factor, using the below equation:


$$r_{\%}=\frac{\left|r_{i}\right|*100}{\sum_{i=1}^{n}\left|r_{i}\right|}$$

In which:

*n:* is the size of the statistics file;

|*r*_*i*_|: is the absolute value of the correlation coefficient.

Subsequently, we selected factors higher than the established threshold significance factor, which we set as above 10% of the correlation share.

## Results

**FIGURE 2:**
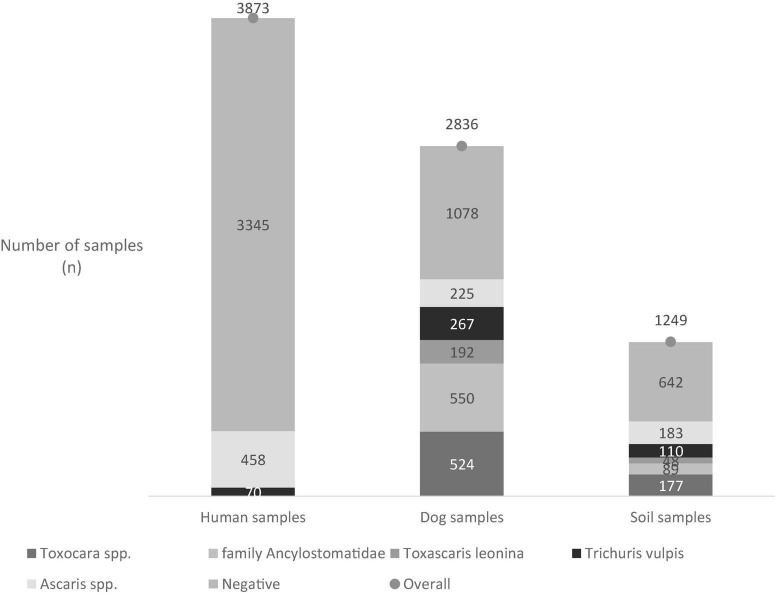
The occurrence of soil-transmitted helminths (STH) in different types of samples.

Overall, 3,873 stool samples were examined for the presence of STH eggs. The overall prevalence was 13.40% (519/3873). The most prevalent taxa represented were *Ascaris lumbricoides* (11.82%; 458/3873) and *Trichuris trichiura* (1.81%; 70/3873; Figure 2).

Subsequently, the studied population was divided according to ethnicity into two groups. The highest rates of STH eggs were found in the minority Roma group, where the prevalence of STH was 25.25%. Roma individuals were infected more often than people from the major population (OR: 37.12, Table 1). The STH eggs prevalence among the majority (non-Roma) group was 0.90%. Comparable results were obtained in the occurrence of *A. lumbricoides* and *T. trichiura* (Table 1).

**TABLE 1:** Soil-transmitted helminths (STH) prevalence in the population of Eastern Slovakia (Košice and Prešov regions) categorized by minority or majority status.

		Total	Positive	Prevalence (%)	95% CI	OR	95% CI
Overall	Minority	1988	502	25.25	5.66–28.96	37.12	22.78–60.48
	Majority*	1885	17	0.90	0–1.90		
*Ascaris lumbricoides*	Minority	1988	443	22.28	5.18–25.37	35.74	21.27–60.06
	Majority*	1885	15	0.80	0–1.69		
*Trichuris trichiura*	Minority	1988	69	3.47	0.79–0.97	67.74	9.4–488.27
	Majority*	1885	1	0.05	0–0.17		

95% CI–95% Confidence Interval; OR, Odds Ratio; *-reference group for calculating OR.

The occurrence of helminth infections in eastern Slovakia Roma and non-Roma populations according to ecosystems (villages, towns) is summarized in Tables 2A, B. In Roma living in villages, the STH eggs were present more often than in Roma residing in cities, where the overall occurrence was 32.79 and 17.94%, respectively (OR: 2.23). The difference between the prevalence of *A. lumbricoides* in Roma living in villages and towns was clearly determined. The infection prevalence in Roma-residing villages was 27.67%. Meanwhile, in cities, it was 16.95% (Table 2A). However, the *T. trichuris* occurrence in Roma from villages and towns was analogous (Table 2A), 3.06 and 3.07%, respectively. The STH eggs prevalence in non-Roma people was less than 1.50% in villages and 0.67% in towns (Table 2B).

**TABLE 2A:** Soil-transmitted helminths (STH) prevalence in the Roma population of Eastern Slovakia (Košice and Prešov regions) categorized by residency type.

		Total	Positive	Prevalence (%)	95% CI	OR	95% CI
Total	Villages	979	321	32.79	0–30.66	2.23	1.81–2.75
	Towns*	1009	181	17.94	4.88–40.36		
*Ascaris lumbricoides*	Villages	979	271	27.68	0–25.91	1.87	1.51–2.33
	Towns*	1009	171	16.95	3.81–38.93		
*Trichuris trichiura*	Villages	979	30	3.06	1.32–8.43	0.78	0.48–1.28
	Towns*	1009	31	3.07	0.02–3.82		

95% CI–95% Confidence Interval; OR, Odds Ratio; *-reference group for calculating OR.

**TABLE 2B:** Soil-transmitted helminths (STH) prevalence in the non-Roma population of Eastern Slovakia (Košice and Prešov regions) categorized by residency type.

		Total	Positive	Prevalence (%)	95% CI	OR	95% CI
Total	Villages	697	9	1.29	0–2.99	1.92	0.74–5.02
	Towns*	1188	8	0.67	0–1.88		
*Ascaris lumbricoides*	Villages	697	8	1.15	0–2.61	1.95	0.71–5.43
	Towns*	1188	7	0.59	0.1.87		
*Trichuris trichiura*	Villages	697	1	0.14	0–0.42	–	
	Towns*	1188	0	0	0		

95% CI–95% Confidence Interval; OR, Odds Ratio; *-reference group for calculating OR; –, unable to calculate due to lack of data.

Out of 2,836 examined samples of dog feces, 43.55% of the samples contained STH eggs. STH infections were present in dogs from villages, towns, and segregated settlements where considerable differences in the prevalence and STH species spectrum were observed. The highest prevalence of infection was detected in dogs from settlements (77.55%), while the prevalence among dogs from the villages and towns was lower (32.22 and 36.68%). Five different STH species were identified. In particular, the *Toxocara canis* eggs, eggs from the family Ancylostomatidae, eggs of *Toxascaris leonina*, *Trichuris vulpis* eggs, and *Ascaris* spp. eggs were present (Figure 2). The findings of *Ascaris* spp. eggs not specific to dogs, especially those living with people in the settlements, were alarming (OR: 232.30). The occurrence of STH eggs in villages, towns, and settlements is summarized in Table 3.

**TABLE 3:** Soil-transmitted helminths (STH) prevalence in the dogs’ excrement is categorized by the environment.

		Total	Positive	Prevalence (%)	95% CI	OR	95% CI
Total	Villages	372	131	35.22	24.04–42.74	0.94	0.75–1.18
	Towns*	1974	724	36.68	26.60–52.16		
	Settlements**	490	380	77.55	–	6.02	4.78–7.57
*Toxocara canis*	Villages	372	62	16.67	20.01–53.93	1.24	0.94–1.72
	Towns*	1974	268	13.58	7.77–20.26		
	Settlements**	490	194	39.59	–	4.01	3.23–4.97
family Ancylostomatidae	Villages	372	41	11.02	3.49–47.77	0.91	0.64–1.29
	Towns*	1974	237	12.01	3.99–25.79		
	Settlements**	490	272	55.51	–	9.28	7.47–11.54
*Toxascaris leonina*	Villages	372	8	2.15	0–12.07	0.48	0.23–0.99
	Towns*	1974	87	4.41	0–15.14		
	Settlements**	490	97	19.80	–	5.85	4.32–7.91
*Trichuris vulpis*	Villages	372	27	7.26	0–49.04	0.95	0.61–1.49
	Towns*	1974	133	6.74	1.33–15.69		
	Settlements**	490	110	22.45	–	4.04	3.1–5.28
*Ascaris* spp.	Villages	372	4	1.08	0–49.04	5.35	1.33–21.5
	Towns*	1974	4	0.20	0–0.82		
	Settlements**	490	217	44.29	–	232.30	113.45–475.66

95% CI–95% Confidence Interval; OR, Odds Ratio; *-reference group for calculating OR in villages. **-reference group for calculating OR in settlements were towns and villages combined; –, unable to calculate due to lack of data.

The high STH eggs prevalence in people and dogs presents a risk factor for disseminating parasitic propagative stages into the environment. Therefore, soil contamination was also cautiously monitored. Thus, the STH eggs presence was assessed in 1,249 soil samples collected from all studied localities, particularly from areas with frequent dog movement. The STH eggs were present in 26.26% of the analyzed samples. In soil from villages, a 33.33% STH eggs prevalence was documented compared to towns, where only 9.48% positivity was observed. The highest STH eggs prevalence (66.96%) in soil was recorded in the segregated settlements (Table 4). The odds of contracting the STH eggs in settlements were 15.94 times higher when compared to urban or rural areas. As shown in Table 4, the eggs of *Toxocara* spp., *T. leonina, Trichuris* spp., and eggs from the family Ancylostomatidae were presented in all researched localities (Figure 2, Table 4). The exception was observed for *Ascaris* spp. eggs, which were present only in the settlement’s soil.

**TABLE 4:** Soil-transmitted helminths (STH) eggs prevalence in the soil is categorized by the environment.

		Total	Positive	Prevalence (%)	95% CI	OR	95% CI
Total	Villages	69	23	33.33	22.68–81.95	4.78	2.75–8.28
	Towns*	844	80	9.48	1.46–10.37		
	Settlements**	336	225	66.96	51.73–79–74	15.94	11.92–21.65
*Toxocara* spp.	Villages	69	7	10.14	0–27.9	2.39	1.02–5.58
	Towns*	844	38	4.50	0.08–3.08		
	Settlements**	336	132	39.29	27.19–50.62	12.48	8.61–18.09
Family ancylostomatidae	Villages	69	12	17.39	0–77.96	5.52	2.69–11.33
	Towns*	844	31	3.67	0–4.26		
	Settlements**	336	46	13.69	0–35.99	3.21	2.07–4.97
*Toxascaris leonina*	Villages	69	7	10.14	0–10.76	23.7	6.76–83.19
	Towns*	844	4	0.47	0–0.51		
	Settlements**	336	37	11.01	2.59–21.23	10.11	5.11–20.14
*Trichuris* spp.	Villages	69	2	2.90	0–20.48	12.56	1.74–90.63
	Towns*	844	2	0.24	0–1.24		
	Settlements**	336	102	30.36	21.77–41.92	99.06	36.1–271.79
*Ascaris* spp.	Villages	69	0	0	–	–	
	Towns*	844	0	0	–	–	
	Settlements**	336	183	54.46	–	–	

95% CI–95% Confidence Interval; OR, Odds Ratio; *-reference group for calculating OR; **-reference group for calculating OR in settlements were towns and villages combined; –, unable to estimate due to lack of data.

**FIGURE 3:**
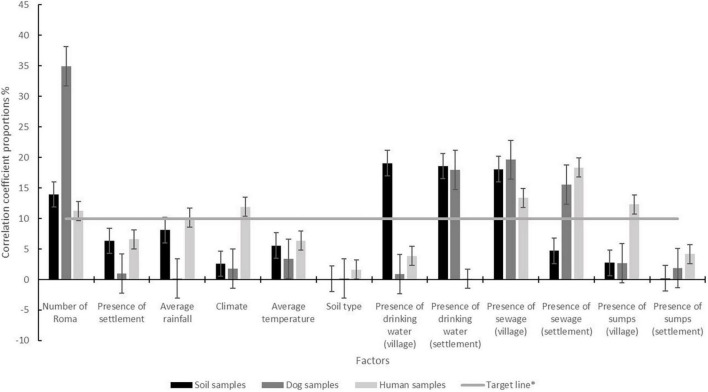
Comparison of correlation coefficients for each factor in percentage in surveyed sample types. Error bars represent standard error. *-Threshold value of 10% of the proportion of the correlation coefficient.

In total, we investigated 12 various factors, which were evaluated and categorized into demographic, climatic, edaphic, and WASH conditions (number of Roma inhabitants, presence of settlements, yearly average rainfall, climate, yearly average temperature, soil type, presence of drinking water in villages and settlements, the existence of sewerage systems, and presence of sumps in villages, towns, and settlements). As shown in Figure 3, out of these factors, poor hygienic conditions, access to drinking water, usage of sewerage, usage of sumps, and climate were the five factors that have a correlation share above 10%. Those were established as our target line and considered significant (Figure 3, Table 5). Similarly, the distribution of STHs in dogs and soil was mainly affected by the denseness of people living in poor hygienic conditions who had limited access to drinking water, and the opportunity for sewerage usage (Figure 3, Table 5).

**TABLE 5:** The established correlation coefficients between the relationship of individual environmental, demographic, and climatic data and type of sample (human, dog, and soil sample).

	Sample type		
	Soil (*r*)	Dog (*r*)	Human (*r*)
Number of Roma	0.465	0.761	0.189
Presence of settlement	–0.212	0.022	0.111
Average rainfall	0.270	0.003	–0.171
Climate	0.086	0.040	0.201
Average temperature	–0.186	–0.074	0.107
Soil type	0.004	–0.004	0.027
Presence of drinking water (village)	–0.637	0.019	0.065
Presence of drinking water (settlement)	–0.620	–0.390	0.003
Presence of sewage (village)	–0.603	–0.427	0.225
Presence of sewage (settlement)	–0.157	–0.338	–0.309
Presence of sumps (village)	0.091	–0.058	–0.207
Presence of sumps (settlement)	0.007	0.040	–0.070

*r*–correlation coefficient.

## Discussion

The term STH refers to a group of particular parasitic human nematodes represented by a roundworm (*Ascaris lumbricoides*), whipworm (*Trichuris trichiura*), and hookworm (*Ancylostoma duodenale, Necator americanus*). According to WHO data, more than 1.5 billion people have become infected by STH worldwide (13). STH occurs not only in developing but also in industrialized countries, including Slovakia. They are especially prevalent in countries where climatic factors and poor hygienic conditions facilitate transmission (14, 15). In our study, the prevalence of STH among inhabitants of east Slovakia was 13.40%. In this survey, most people affected by STH–*A. lumbricoides* and *T. trichiura* belong to the minority (Roma). This was caused primarily due to the constant interpersonal contact with sources of contamination and hygienic habits. It is known that Roma inhabits shacks and houses in the villages, apartments with reduced living standards found, or abandoned homes in urban areas. In the cities, Roma settled in areas commonly called ghettos (3, 16, 17).

Examination of 1988 Roma and 1885 non-Roma fecal samples led to the conclusion that the risk of STH infection is 37.12 times higher among the Roma inhabitants compared to the non-Roma population. Infections with *A. lumbricoides* were observed more frequently than with *T. trichiura.* Undoubtedly because of the presence of more resilient sticky eggs, which embryonated more rapidly, they can survive for years and may be more uniformly distributed in the environment, thus increasing the risk of infection (18, 19). On the other hand, Chis Ster et al. (20) observed strong associations between *T*. *trichiura* infections and people living in the poorest households with infected siblings. Sharing a poor household with infected siblings, who instantly contaminate the peri-domestic environment frequently with *T*. *trichiura* eggs maximizes the chance of transmission.

In Northeastern Slovakia, Pipiková et al. (3) reported helminth ova in 53.17% of stool samples of Roma children, where the eggs of *A. lumbricoides*, *T. trichiura*, and *Hymenolepis diminuta* were the most abundant. In contrast, the helminth ova were not seen in the non-Roma children population living in the localities where common hygiene standards are followed. Intestinal helminth infections among children living in eastern Slovakia were also examined by Pipiková et al. (2). According to this study, the infection prevalence was 16.90%. The most prevalent nematode species was *A. lumbricoides* (14.32%), followed by *T. trichiura* (3.76%), *Hymenolepis nana* (0.94%), and *H. diminuta* (0.23%). The infection prevalence among children from the majority (non-Roma) group was only 0.66%. Meanwhile, the infection prevalence among children from the minority (Roma) was 25.82%. The previous study results of Königová et al. (21) correspond with our data, where intestinal helminths were found in 2.55% of children in Slovakia. Again, the most prevalent were *A. lumbricoides* and *T. trichiura*, but data regarding the residence and nationality of participants were absent in this study. The results of Rudohradská et al. (1) correspond with our data, where 56.8% of Roma children were positive for the presence of intestinal parasites and *A. lumbricoides* and *T. trichiura* was the leading parasite. The crucial determinants of STH infection spread are ethnicity, lower educational level, household overcrowding, and environmental factors indicative of greater poverty and marginalization associated with STH infections. Improvements in living conditions of Roma populations can lead to significant reductions in STH infection spread risk.

Since the soil and domestic animals are the primary media for soil-transmitted helminthiasis spread, they were also examined for the presence of the STH eggs to show the environmental burden. In general, the presence of STH eggs was confirmed in 26.26% of all soil samples. The detailed description is as follows: *Toxocara* spp. eggs (14.17%), *Trichuris* spp. (8.49%), eggs from the family Ancylostomatidae (7.16%), and eggs from *Toxascaris leonina* (3.84%). *Ascari*s eggs (14.65%) were detected only in the soil from localities with low hygienic standards. Compared to urban or rural areas, the probability of contracting STH eggs in segregated settlements of marginalized groups was 15.94 times higher. Our results correspond with those of Wahyuni (22). He reported that a supporting factor for the high prevalence of STH is high population density localities with a distance between houses lesser than 1–2 meters so that sunlight cannot reach the ground. As a result, the soil around the settlement becomes permanently moist. David et al. (23) reported that STH infections in endemic areas are spread not only due to unhygienic and poor living conditions but also due to environmental factors allowing the dispersion and development of STH infective stages. *A. lumbricoides* eggs are more resistant to environmental conditions and can remain viable in the soil for several months by undergoing aestivation, permitting survival (19, 24). The high occurrence and distribution of the STH may also be related to the physicochemical properties of the soil, adequate rainfall, ambient soil temperature, and soil pH range close to neutral (25).

The primary sources of the infected environment are feces from infected animals living in close vicinity to the man. Contact with an animal is more intense in rural than urban ecosystems. The likelihood of diseases spreading is even more significant by stray animals or animals without veterinary control. On the contrary, the town’s infrastructure with flats and apartments impacts how dogs are kept and share their living space with owners. Dogs in cities are in close contact with humans, have become part of households, and may contribute to infection spread. Our study confirmed the STH eggs in 43.55% of dog droppings, predominantly of *T. canis* eggs (18.48%), *T. vulpis* (9.52%), eggs from the family Ancylostomatidae (19.39%), and *T. leonina* eggs (6.77%). The most noteworthy finding was that eggs of the genus *Ascaris* represented 7.93% of dog droppings from localities with a low hygienic standard were positive. The presence of *Ascaris* spp. provides evidence of the dogs’ coprophagous habits where ascariasis spreads primarily by fecal contamination of the environment around human dwellings because of defecation outside the toilets.

The highest prevalence of infection was detected in dogs from settlements (77.55%), while the prevalence among dogs from the villages and towns was lower (32.22 and 36.68%). Our results correspond with those published by Antolová et al. (26) and Szabová et al. (27). Papajová and Šoltys (5) reported the total prevalence of intestinal nematodes in dogs from urban localities in Slovakia at 25.45% and in dogs from rural localities at 40.09%. *T. canis* and the eggs from the family Ancylostomatidae appeared to be the most frequent. The prevalence of intestinal parasites among dogs from areas with low environmental hygiene was 80.59%, and 13 helminthic species with the predominance of *Ascaris* spp. and the family Ancylostomatidae were identified (5). Rudohradská et al. (28) reported a high parasitic incidence in dogs from segregated Roma settlements in Slovakia. The eggs of at least one parasite were detected in 73.8% of dog feces samples. Pipiková et al. (3) also confirmed differences in the prevalence and species diversity of detected helminths in dogs from two northeastern Slovakian localities with different hygiene standards. Parasitic infections were present in both study areas. Still, fewer parasitic infections in dogs and less contaminated soil were found in the village with a higher standard of living, better personal and communal hygiene levels, and better animal care. On the contrary, the total prevalence of intestinal parasites among dogs from the village where the Roma minority lives were 71.65%, and 12 different taxa with the predominance of the family Ancylostomatidae and *Ascaris* spp. were identified. But the prevalence of intestinal parasites in dogs from the village with better hygiene was 19.44%, and only four taxa (*T. leonina* and *T. canis, Sarcocystis* spp., and *T. vulpis*) were detected (3).

Several environmental factors influence the occurrence of STH infection. First, variables such as demographic, climatic, edaphic, and WASH conditions were utilized in this study. Due to the importance of environmental factors and WASH (water, sanitation, and hygiene) conditions in the transmission processes, it was highly relevant to establish a relationship between the environmental risk factors and spatial patterns of STH infections. Twelve factors were evaluated and categorized as mentioned above. The descriptive statistics and correlation coefficient was used to assess the effectiveness of variables modeling STH distribution. The calculated correlation coefficient told us which factors influenced the spread of the diseases. Its values are generally in the range of −1 to +1. The 10% selection results from its definition that a correlation coefficient closer to 0 indicates that there is no or weak correlation (12). So, this value was chosen to consider the factors that are important to us. Since each factor affects the occurrence of STH in the environment to a certain extent. The determination of the upper limit of the correlation weight is not very objective. Out of the 12 factors investigated, poor hygienic conditions, access to drinking water, usage of sewerage, usage of sumps, and climate were the five most important factors responsible for the STH distributamongn among inhabitants. The distribution of STHs in dogs and soil was mainly affected by the denseness of people living in poor hygienic conditions having limited access to drinking water, and the opportunity for sewerage usage. The statistical results show that poor sanitary conditions, access to drinking water, use of sewerage, usage of sumps, and climate play an essential role in the STH distribution among people. The distribution of STHs by dogs and soil was affected by the number of people living in poor hygienic conditions where access to drinking water and sewerage usage were the other important contributing factors.

Our results correspond with those of Scholte et al. (15), where it is shown that factors associated with the transmission of ascariasis and trichuriasis are related to environmental variables and the poverty index. Massara and Enk (29) also confirmed the relevance of basic sanitation (proper treatment of effluents and potable water supply) and health education are measures that could reduce the probability of *Ascaris lumbricoides* infection. Yaro et al. (30) stated that various climatic and edaphic factors influence the distribution of STHs in Africa. The top five most important factors responsible for their distribution were the mean diurnal temperature range, annual temperature range, the maximum temperature of the warmest month, precipitation of the wettest quarter, and soil clay contents. Campbell et al. (31), Ngaluma et al. (32), and Spada and Asmalinda (33) confirmed that such factors are tropical and subtropical climates (high humidity and hot temperatures), soil type (warm and wet), soil particle properties, and soil cultivation methods. According to Wahyuni (22), Noviastuti (34), Da Silva, et al. (35), Wardell et al. (36), Riaz et al. (37), Saskyarasmi et al. (38), the distribution of the STH in areas with lower hygiene and sanitation standards is closely related to the following: the environmental sanitation factor (toilets, latrines, sewage treatment, personal hygiene), defecation facilities (defecating openly on the ground, using open latrines in rivers), houses with dirt floors, high population density, sources of drinking water, food contaminated with STH eggs. According to Wardell et al. (36), Wardell et al. (39), Pullan et al. (40), and Alvarez di Fino et al. (41), the most critical factors affecting STH are the ranges of temperature and rainfall precipitation.

From evaluating the current epidemiological situation in Eastern Slovakia, we can conclude that ethnicity and hygiene are the most important factors in the distribution of STH infection. Also, cultural-living habits appear to be a causal factor in the STH spread. This study was the first large epidemiological study pointing to this phenomenon which will be examined in the future with ontology experts’ help on Roma culture.

Soil-transmitted helminthes (STH) infections continue to be a public health great concern. However, its monitoring remains a major challenge. Partially due to a shortage of samples gathered in studies where the sample return rate is frequently low. Additionally, the Epidemiological Information System of Slovakia (EPIS) lacks information on the prevalence of STH. Therefore, this study attempts to raise public awareness of regretful health and epidemiological situation. The novelty of this study distinguishes it from other studies dealing with related subjects that have been undertaken in Slovakia. This is the first publication dealing with identifying the risk factors for STH transmissions in Slovakia. The previous research papers did not evaluate the risk areas based on various environmental factors influencing the spread of STH. Our study, in contrast to others like Pipikova et al. (1) (which only examined human samples), deals with a variety of sample types (dogs, humans, and soil samples). The sample size is in the thousands of samples examined. This is in contrast to other studies where only a few hundred samples were examined [Pipikova et al. (2)]. Another strength is the sizeable territorial focus, which has yet to be fully completed in Slovakia. The study by Papajova and Šoltys (5) has already addressed several points but still fell short on the magnitude of the research. However, we are aware that our study is limited by examining only one stool sample per participant or animal. A lack of cooperation with parents, legal guardians, and dog owners, likely may have led to an underestimation of the ‘true’ prevalence. Also, in some studied localities, soil sampling was impossible due to the intimidating behavior of its inhabitants. Finally, we believe that the fundamental governmental institutions will establish suitable safeguards for urban and rural environments. This would need more educational presentations in schools for the younger generation or informative leaflets about STH, as well as the introduction of various grants from the state or European union to municipalities to acquire essential sanitary items, construct latrines, or even install sewage systems, thus improving the level of protection. Expanding veterinary services for stray dog catching and household animal dehelminitization should be a primary step to prevent STH spread.

## Data availability statement

The raw data supporting the conclusions of this article will be made available by the authors, without undue reservation.

## Ethics statement

The studies involving human participants were reviewed and approved by the Ethical Commission of Košice Self-governing Region (Document no. 5436/2019/ODDZ-25820). Written informed consent to participate in this study was provided by the participants’ legal guardian/next of kin. This animal study was reviewed and approved by the research related to animals complied with all the relevant national regulations and institutional policies for the care and use of animals. Written informed consent was obtained from the owners for the participation of their animals in this study.

## Author contributions

All authors listed have made a substantial, direct, and intellectual contribution to the work, and approved it for publication.
